# Right-hemisphere lateralisation evidenced from the chimeric face task predicts self-reported social competencies

**DOI:** 10.3758/s13415-025-01378-x

**Published:** 2025-12-10

**Authors:** Vinh Nguyen, Robin Laycock

**Affiliations:** Psychology Department, School of Health & Biomedical Sciences, Building 202, Level 4, 264 Plenty Rd, Mill Park, Victoria, 3082 Australia

**Keywords:** Hemispheric lateralisation, Right hemisphere superiority, Face perception, Social perception, Social competencies

## Abstract

Observing and understanding faces is a critical component of social interactions. The neural correlates of face processing have been well established to be preferentially lateralised to the right hemisphere, though the functional role of this brain asymmetry has received less attention. Here we investigated the hypothesis that a left-visual-field (right-hemisphere) bias in face perception would be associated with a broader set of self-reported social competencies. Participants (*n* = 348) completed a chimeric face task, requiring judgements of which side of chimeric face stimuli were more emotional, a face emotion recognition task, and the Multidimensional Social Competence Scale in an online experiment. Overall social competencies were predicted by degree of chimeric face task bias to the right hemisphere (determined as the laterality quotient, LQ). Structural Equation Model analyses revealed that social inferencing and non-verbal sending skills were best predicted by LQ. In all analyses the predictive role of LQ was independent of face emotion recognition. Social cognition has previously been linked to the right hemisphere, but we report a novel relationship between lateralisation of face processing, and aspects of social competencies that encompass both understanding and the display of social cues.

**Supplementary information:**

The online version contains supplementary material available at 10.3758/s13415-025-01378-x.

## Chimeric face task-based right-hemisphere lateralisation predicts social competencies

Humans are social beings with a need to form and maintain interpersonal relationships. This outcome is contingent on a range of social competencies, in particular the ability to perceive and interpret socially relevant information (Baumeister & Leary, 1995). Social competencies can include turn taking in conversation, understanding social cues, and the incorporation of social norms, which allow an individual to dynamically engage in successful interpersonal interactions (Trevisan et al., 2018). The proficiency of these social competencies is an important predictor of multiple indicators of social and emotional success. For example, social-emotional skills in kindergarten children can predict later education, employment, and mental health outcomes (Jones et al., 2015).

One important component of social-cognitive competencies is the ability to make inferences about another person’s emotions, intentions, and behaviours from their facial expressions (Horstmann, 2003). Indeed, face processing is developmentally important, with evidence that increased attention to faces at 7 months predicted prosocial behaviours at 2 to 3 years of age (Peltola et al., 2018). The developmental course (Pascalis et al., 2011) and the neural correlates of face processing (Batty & Taylor, 2006; Leppänen & Nelson, 2009; Scherf et al., 2007) have been well characterised. Indeed, infants as young as one hour show a preference for looking at faces, a mechanism likely driven more by subcortical processes (Johnson, 2005; Johnson et al., 1991), while the cortical system involved with face processing develops throughout early childhood into adolescence (Cohen Kadosh et al., 2010; Scherf et al., 2007).

There is now overwhelming evidence that the right hemisphere preferentially processes face information, as demonstrated from adult studies using neuroimaging (Kanwisher et al., 1997), event-related potentials (ERPs; Caharel et al., 2009; Jacques et al., 2007; Rossion et al., 2003b) and neuropsychological evidence, with unilateral brain damaged patients showing impaired face perception following right hemisphere damage (Abbott et al., 2013) but not the left hemisphere (Adolphs et al., 1996; Rossion et al., 2003a). Behavioural measures also support the predominance of the right hemisphere for face processing (Bourne & Gray, 2011; Indersmitten & Gur, 2003; Innes et al., 2016). These behavioural measures suggest there could be some development of this bias throughout childhood (Aljuhanay et al., 2010; Workman et al., 2006), with functional evidence showing right-hemisphere dominance for faces in 4- to 6-month-old babies using electroencephalography (de Haan & Nelson, 1999; de Heering & Rossion, 2015). In fact, newborn infants already demonstrated face discrimination in right but not left hemisphere ERPs following presentation of faces to each hemifield, with development of this response found only in the right hemisphere from 5 to 25 weeks (Adibpour et al., 2018). Furthermore, infants who had been deprived of visual input to the right, but not the left, hemisphere, showed severely impaired face processing (Grand et al., 2003). Together these findings signify a crucial neurodevelopmental period for hemispheric lateralisation of face processing.

The functional utility of such lateralisation is less clear (Rossion & Lochy, 2022). In general terms, cerebral lateralisation has been suggested to confer advantages in terms of neural efficiency in performing cognitive tasks (Rogers et al., 2004). However, it is still not clear whether brain asymmetries, for example in language, serve an evolutionary advantage or instead are a legacy of earlier evolutionary developments on brain organisation (Dräger et al., 2005; Vallortigara et al., 1999). In the current study, given the important role of face perception in facilitating social interactions, and in social cognition more broadly, we consider the possibility that a right hemisphere bias for face processing is linked to a broader set of social competencies. For example, in frontotemporal dementia, which is linked to degeneration of social behaviour, diffusion tensor imaging demonstrated that deficits in detecting sarcasm in social situations was linked with frontotemporal white matter tract changes in the right hemisphere (Downey et al., 2015).

The most prevalent behavioural measure of face lateralisation is the chimeric faces task (Butler et al., 2005; Harrison & Strother, 2021; Innes et al., 2016; Levy et al., 1972, Workman et al., 2006). Chimeric faces are images created by positioning two half faces that differ on some construct, for example emotion, to create a single face. Commonly, chimeric face tasks involve presenting the mirror image of two chimeric faces with participants required to judge with face is more emotional. A perceptual bias in selecting the face displaying the emotional expression on the left (i.e., a left visual field bias) is taken as an index of right hemisphere dominance (Innes et al., 2016). If an association between strength of right-hemisphere bias and a given cognitive function were established, this could imply a functional role for this lateralisation. We note that the chimeric face paradigm can be utilised in the context of emotion research, for example to support the Right Hemisphere Hypothesis of emotion (Borod, 1992; Bourne, 2010). Although there is conceptual overlap between emotion and facial emotion perception, chimeric face paradigms have not focussed exclusively on emotion; chimeric faces split by identity (Harrison & Strother, 2021; Yovel et al., 2008) and gender (Aljuhanay et al., 2010; Butler et al., 2005) have also replicated the left visual-field bias in face perception. Given the complexity of emotion as a construct consisting of perception, experience, and the expression of emotion (Alves et al., 2008), we limit our primary focus to face perception, acknowledging the conceptual link to face emotion perception.

The possibility of an association between lateralisation of face processing and social competencies, emerges from both correlational and experimental evidence. Workman et al. (2006) demonstrated in children that the strength of the right-hemisphere bias in the chimeric face task corelated with ability to recognise emotions from cartoon scenarios and pictures of the eye region of faces. Similarly, Watling & Damaskinou (2020) revealed that the degree of right-hemisphere bias for face perception at 12 years of age predicted emotion matching ability 1 year later. There is also some evidence for a reduced right-hemisphere bias for face emotion processing in autistic populations, providing a further link between face lateralisation and social competencies. For example, using a chimeric face task Taylor et al. (2012) found that autistic children aged 11–15 years demonstrated no lateralisation for most emotions, and only a right-hemisphere bias for happy and angry faces. As the authors noted, this pattern of results was similar to that observed in 5- to 6-year-old nonautistic children, and less lateralised than 7- to 8-year-old’s as reported in a previous study (Workman et al., 2006), suggestive of a developmental delay. Similarly, Ashwin et al. (2005) found evidence of reduced right-hemisphere bias for an identity- but not an emotion-based chimeric face task in adults with Asperger’s, suggesting that the link between lateralisation measured by the Chimeric task and social-communication skills may be related more to face lateralisation rather than emotion lateralisation.

Babies with an increased likelihood of being autistic (Dundas et al., 2012b), as well as autistic adults (Dundas et al., 2012a) tend to fixate less on the left side of a face, while MEG data revealed reduced right temporal lobe activation in autistic adolescents during an implicit face emotion task (Leung et al., 2015). Finally, there is also evidence to suggest that variation in lateralisation of face processing extends into the nonautistic population. Dimensional autistic traits in neurotypical adults predicted lateralisation in a chimeric faces task, with higher autistic traits associated with reduced right-hemisphere bias in females, but predicting a stronger right-hemisphere bias in males (Vladeanu et al., 2012).

Given evidence that the right hemisphere is associated with prosocial tendencies (Hecht, 2014), and that deficits in social cognition are associated with abnormal right hemisphere structure and function in neurodevelopment (Taylor et al., 2012) and in neurological conditions (Downey et al., 2015), the current study sought to investigate whether degree of lateralisation of face processing, over and above face emotion recognition accuracy, was predictive of variation in social competencies in a community sample. Importantly, there is already some evidence that right-hemisphere bias for face processing predicts other face-related emotion processing (Watling & Damaskinou, 2020; Workman et al., 2006) and has also been linked to lower trait anxiety but higher social anxiety (Bourne & Vladeanu, 2011). However, to our knowledge, only one study has examined any broader aspects of social cognition in the context of assessing lateralisation of face processing. In this study, a positive association was established between a task designed to assess awareness of emotional experiences and face lateralisation as assessed with a chimeric face emotion task (Lane et al., 1995). We significantly extend on this by assessing a much broader set of competencies relating to social communication and how this could be related to lateralisation of face processing. In order to address whether face lateralisation was a predictor of social competencies over and above emotion recognition, we also included a measure of face emotion recognition. It was hypothesised that a right-hemisphere bias would be associated with both increased face emotion recognition and with stronger social competencies. Furthermore, as the relative subfactors of social competencies as suggested by the Multidimensional Social Competence Scale (MSCS) do not have an established face validity within the wider literature and may further provide specific commentary on the facets of social competency, we also aim to more widely explore the relationship between face lateralisation and the subfactors of the MSCS.

## Methods

### Participants

Participants were 411 adults who voluntarily consented to participate and received partial course credit for participation. Experimental procedures were approved by the College Human Ethics Advisory Network at RMIT University. Only those indicating right handedness were included given this could influence face lateralisation (Bourne, 2008), resulting in the exclusion of 33 left-handed participants, while 12 participants were excluded after indicating needing corrective glasses but to not be wearing them currently. This resulted in a sample of 366 adults with a mean age of 20.6 (*SD* = 2.8, range 18–34), including 266 female, 89 male, and 11 nonbinary identifying participants. Eighty-six participants indicated the existence of a mental health condition, most commonly including anxiety, depression, and ADHD. Using G*Power (version 3.1.9.7; Faul et al., 2009), the current sample fulfills the *a priori* power (.8, α =.05) to find a small to moderate effect size in a simple regression analysis (*F*^2^ =.03, recommended *n* > 325). Additionally, a set of power analyses were conducted for structural equation modelling at the item level, with a sample size of 338 suitable to avoid model misspecification based on an RMSEA of.05 using pwrSEM (version 0.1.2; Wang & Rhemtulla, 2021), assuming degrees of freedom at 2,886, given 77 items, measurements for chimeric LQ and facial emotion recognition (FER) accuracy and nine latent variables (Collier, 2020). A series of Monte-Carlo 1000 simulations of the model showed that power exceeded.8 for the parameter estimates for the SEM model with a hypothetical sample size of 338, assuming a medium parameter estimate of.35,.75 for item factor loadings and.51 for covariates with values suggested by Colliler (2020), also utilising pwrSEM.

## Materials

### Social competencies

To assess social competencies, we utilised the Multidimensional Social Competence Scale (MSCS; Yager & Iarocci, 2013). The MSCS was originally developed as a parent-report scale for adolescents but was more recently validated as a self-report scale for adults suitable for online administration (Trevisan et al., 2018). The MSCS comprises 77 items, with 11 items in each of seven domains: Social Motivation (SM), Social Inferencing (SI), Demonstrating Empathic Concern (DEC), Social Knowledge (SK), Verbal Conversational Skills (VCS), Nonverbal Sending Skills (NSS; Trevisan et al. sometimes referred this as Nonverbal Conversation Skills), and Emotional Regulation (ER). Answers are provided using a 5-point Likert scale, ranging from (1) not true or almost never true, (2) rarely true, (3) sometimes true, (4) often true, to (5) very true or almost always true. A higher score on the MSCS indicates superior social competencies. The MSCS also has a suggested two-factor structure in lieu of the total score, with the Social Responsiveness factor summarizing SM, DEC, and NSS, and the Social Understanding factor summarizing SI, SK, VCS, and ER subscales (Trevisan et al., 2018). The MSCS has good convergent validity (as indicated by a correlation of *r* = −.662 with a measure of autistic traits, the autism spectrum quotient), and good internal consistency (Cronbach’s alpha =.795). In the current study Cronbach’s alpha was.947 for the total scale, with subscale values overall similar to Trevisan et al. (2018). See Table 1 for subscale Cronbach’s alpha values for current sample.

**Table 1:** Multidimensional Social Competency Scale factor and subscale internal consistency scores

Scale	Number of items	Cronbach’s α
MSCS total	77	.947
Social responsiveness factor	33	.931
Social motivation	11	.877
Demonstrating empathic concern	11	.871
Nonverbal sending skills	11	.857
Social Understanding Factor	44	.920
Social inferencing	11	.846
Social knowledge	11	.776
Verbal conversation skills	11	.837
Emotional regulation	11	.841

### Chimeric face task

**Fig. 1:**
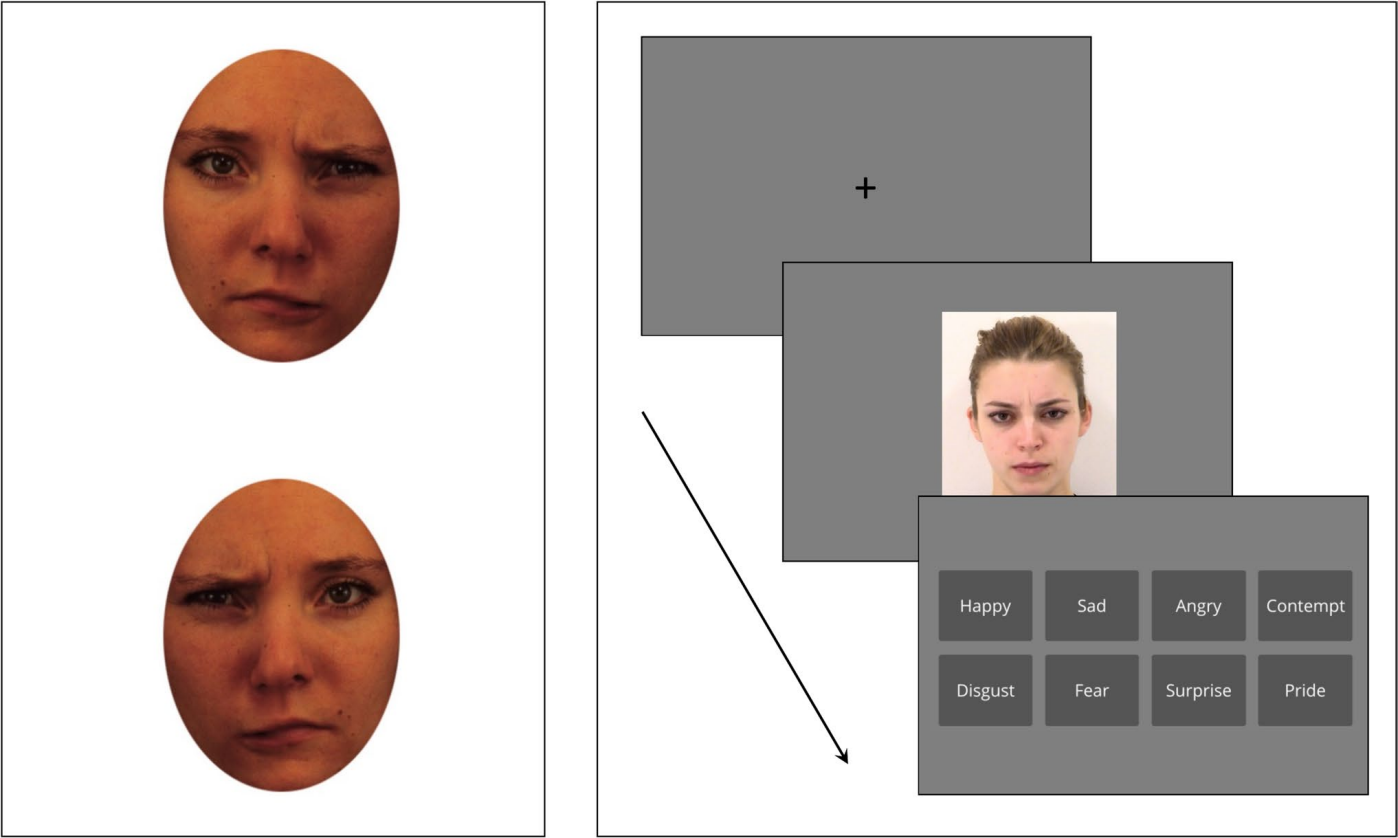
Left: Example display for the chimeric face task. The top image shows a left hemi-face with neutral expression, and the right hemi-face with an angry expression. The lower image is the mirror reversal of the top image. Right: Trial sequence for the face emotion recognition task. Faces were either static or dynamic displays of different emotions

Lateralisation of face processing was measured with a Chimeric Face task. In the version utilised here, chimeric photos were generated from the photos of three male and three female actors from the Karolinska Directed Emotional Faces database (Lundqvist et al., 1998), with each actor providing an exemplar of each expression included. The literature does not provide consensus regarding how many, and which emotions are most appropriate (Bourne & Gray, 2011; Indersmitten & Gur, 2003; Workman et al., 2006), and we selected four expressions to balance the goal of providing a range of emotions, against the practicalities of minimising task duration. Each chimeric face was created by placing the right half of an expressive face, next to the left half of a neutral expression of the same actor. Next, using Adobe Photoshop, a gradient function was used to blur the transition from the neutral to the expressive images, and the images were cropped to an ellipse shape to remove hair and other features around the face. Finally, a duplicate mirrored set of these chimeric faces was generated, creating a set of 24 pairs with the expressive face on the right and the left (Fig. 1).

The chimeric face task involved the presentation of a central black fixation cross for 500 ms on a white background. Subsequently, this was replaced by a pair of chimeric faces presented with one above and one below fixation. The faces were presented until a response was given, and then a blank white screen was shown for 500 ms prior to the next trial. Participants were given written instructions that they would see two faces, and they would need to indicate by pressing the up or down arrow key, which of the pair was most emotive and had the strongest emotion. The task consisted of 48 trials, with each Chimeric face presented above and below the fixation, such that there was an equal probability of the left or right expressive face appearing in each location. The task was scored by subtracting the number of responses to the face with emotion on the left from the number of responses to the face with emotion on the right, divided by the total number of trials. This Laterality Quotient (LQ) provided an index of visual-field bias, with −1 indicating the strongest left-visual-field (i.e., indicating right-hemisphere) bias, and +1 indicating a right visual-field (i.e., indicating a left hemisphere) bias.

### Face emotion recognition task

Stimuli were taken from the Amsterdam Dynamic Facial Expression Set (ADFES; van der Schalk et al., 2011) and consisted of stimuli from four male and four female actors with eight expressions from each actor (angry, sad, fearful, happy, disgust, surprise, contempt, and pride). We included both videos and photographs, as the vast majority of face perception studies have relied on static faces (Dawel et al., 2022), while dynamic presentations providing a more realistic stimulus with greater contextual cues than static images (Dobs et al., 2018). We took an exploratory approach as to whether one or both stimulus types would be related to social competency. A full set of 64 videos was used, with each video edited from the original ADFES set to provide a “partial” expression. Each video consisted of a neutral expression for 500 ms before the dynamic phase whereby the video continued until the expression first emerged (dynamic phase of these edited videos had a mean duration of 275 ms, *SD* = 175 ms). Then, a matched set of 64 photos was created by taking the still image of the last video frame. Participants viewed a 500-ms black fixation cross on a grey background before viewing the static or dynamic face images, which were presented in separate counterbalanced blocks of trials. After stimulus presentation, the screen displayed a set of 8 boxes each with the text of the possible expressions displayed (Fig. 1). Participants had an unlimited time to use a mouse to select their answer, having been informed that they should not spend too long on any one item.

## Procedure

This study was completed online by participants at a time of their own choosing and was created by using the online behavioural experiment builder Gorilla (Anwyl-Irvine et al., 2020). Participants were first provided with a plain language statement and gave consent to participate. Demographic data was collected and subsequently participants completed the Multidimensional Social Competence Scale, and then either the Chimeric Face task or the Face Emotion Recognition Task, which were completed in a counterbalanced order across participants.

## Data cleaning

Because of the online nature of the experiment, as a conservative approach to identify nongenuine or inattentive participants, we first screened the data by excluding participants who either completed the two face tasks too quickly, indicating responses were made without time for a decision, or if participants took too long likely indicating distraction or otherwise not engaging in a focussed manner. For the latter criterion, we calculated the mean task completion time and excluded those greater than 3 standard deviations above the mean. For the Chimeric Face, we deemed it unreasonable to complete the task in less than 1.5 min. Conversely, mean completion time was 3.53 min (*SD* = 1.87), resulting in those taking longer than 9.14 min being excluded. For the FER task, we deemed it unlikely to be genuine if completion time was less than 5 min, whereas the mean (7.91 min, *SD* = 4.48 min) resulted in those taking longer than 21.35 min being excluded. Together, these criteria resulted in the exclusion of 18 participants and a final sample of 348.

## Statistical analyses

The simple statistical analyses were conducted using IBM SPSS Statistics (Version 29). Following this, a series of comparative structural equation models (SEM) were conducted across the items to confirm the factor models outlined in Trevisan et al. (2018), whilst also testing the predictability of LQ onto the subscales of the MSCS. Jamovi (Version 2.6) was used alongside the semlj module (Version 1.2.5), which calls the popular R package for SEM, lavaan (Version 0.6–19; Rosseel, 2012). Item level models were estimated using weighted least square mean and variance-adjusted (similar to diagonally weighted least squares), an estimation method that has better performance than maximum likelihood when considering ordinal data, such as Likert item data (Li, 2016). Conversely for factor totalled data, maximum likelihood was utilized. Latent variables were fixed to the first indicator at 1.

## Results

Despite a strong literature suggesting face processing demonstrates a right-hemisphere bias, there are some different accounts that lateralisation of face emotion processing may depend on the valence or goal-related factors of the emotion (Najt et al., 2013). Hence, we also calculated the LQ score for each emotion. However, for all emotions tested, the current sample demonstrated a significant left-visual-field (indicating right-hemisphere) bias for face emotion perception (Anger LQ: *M* = −0.22, *SD* = 0.46; Fear LQ: *M* = −0.22, *SD* = 0.47; Happy LQ: *M* = −0.15, *SD* = 0.5; Sad LQ: *M* = −0.18, *SD* = 0.43; one-sample t-test against 0, all *p*’s <.001, Cohen’s *d*’s 0.31–0.47). As a final check for the possible contribution of emotion, we also compared the correlation of the LQ for each emotion with the measure of social competencies (MSCS). Correlations for angry, happy, and sad were significant (*r*’s = −.12 to −.13, *p*’s =.016 to.027), while the correlation was not significant for fear (*r* = -.09, *p* =.079). Hence the emotions utilised here are likely to convey similar information about face lateralisation, and in the following analyses we did not consider emotion separately, instead focussing on the combined LQ across all emotions.

Regarding FER performance, participants viewed both static and dynamic partial expressions of emotion, and as expected, accuracy was significantly higher for dynamic compared with static faces, *t*(347) = 13.49, *p* <.001, *d* = 0.72, replicating the “dynamic advantage” phenomenon (Dobs et al., 2018). However, the strength of the correlations between static, dynamic, and combined FER accuracy scores with the LQ, MSCS, and their subscales was remarkably consistent (Table S1). Furthermore, noting that the FER task assessed eight emotions while the Chimeric face task incorporated four emotions, we ran further analyses to assess whether this difference could have influenced the findings. As outlined in the supplementary information, FER accuracy correlated strongly with a revised FER score analysing only the four emotions used in the Chimeric face task, and both FER scores revealed the same pattern of correlations with LQ and MSCS scales (Table S2). Consequently, for the main analyses, we used the combined FER accuracy score, taken as the percentage correct across static and dynamic conditions and all eight emotions.

Descriptive statistics of the sample characteristics, chimeric LQ scores, and MSCS scores are available in Table 2. All items were found to have good internal consistency with the recommended and second-order factorisation (Trevisan et al., 2018), with Cronbach’s α ranging from.776 to.947 (Table 1). Due to a concern that gender differences could be relevant in aspects of face emotion perception or social competency, despite not forming part of the study rationale, independent samples *t* tests were conducted to explore gender differences on all characteristics. A full account of these analyses can be found in the supplementary information. Briefly, we found no gender differences in visual-field bias (LQ), only a small advantage for females in face emotion recognition accuracy (<3%), and no gender difference for total MSCS (Table S3).

**Table 2:** Descriptive statistics for participant characteristics, laterality quotient, FER accuracy, and MSCS scores

	All participants *N *= 348	
	*M*	*SD*
Age	20.61	2.77
Chimeric LQ^a^	−0.19	0.40
Facial Emotion Recognition Accuracy	58.87	8.46
**MSCS total**	289.82	30.48
**Social understanding factor**^**b**^	165.44	17.57
Social motivation	38.26	6.94
Demonstrating empathic concern	44.45	6.08
Nonverbal sending skills	42.55	6.23
**Social responsiveness factor**^**b**^	125.30	16.17
Social inferencing	42.13	5.65
Social knowledge	47.90	4.42
Verbal conversation skills	38.59	6.13
Emotional regulation	36.55	6.89

^a^Chimeric LQ scores closer to −1 indicate increased left-visual-field (indicating right-hemisphere) bias and scores closer to +1 indicate increased right visual-field (indicating left-hemisphere) bias

^b^The 2-factor MSCS model is derived from Trevisan et al. (2018). The social understanding factor consists of social motivation, demonstrating empathic concern and nonverbal sending skills subscales. The social responsiveness factor consists of social inferencing, social knowledge, verbal conversation skills, and emotion regulation subscales

Bolding denotes higher level factors

**Fig. 2:**
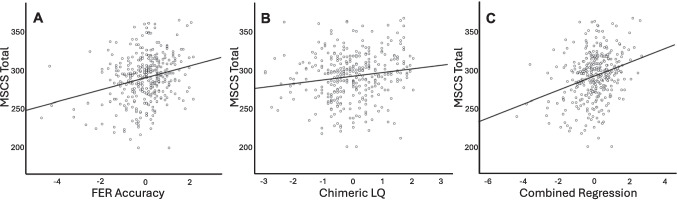
A series of regression model plots, demonstrating a stepwise hierarchical regression (**A**) first with facial emotion recognition (FER) accuracy independently predicting total multidimensional social competency scale (MSCS) scores, (**B**) second with chimeric laterality quotient (LQ) predicting total MSCS scores, (**C**) and finally, with the combined regression for both FER accuracy and chimeric LQ predicting total MSCS scores. All predictor values were standardized to allow for comparison of regression models. Note that the true directional effect of chimeric LQ depicts a negative association (*r* = −.16, *B* = −11.64)

To determine the explained variance of laterality for face processing over FER accuracy on social competency, a hierarchical linear regression was conducted with FER accuracy entered in the first step then chimeric LQ entered in the second step, predicting MSCS total between all participants. This conservative approach was taken despite the absence of a correlation between Chimeric LQ and FER accuracy (*r*(346) = −.03, *p* =.60), however considering that FER correlated significantly with MSCS (*r*(346) =.26, *p* <.001). Facial emotion recognition accuracy significantly but minimally accounted for MSCS total in all participants (*R*^2^ =.06, *p* <.001) and the addition of Chimeric LQ to this model explains 3% more variance (*R*^2^ =.09, *p* <.001). See Figure 2 for a graphical representation, comparing the stepwise models with standardized predictors. Interestingly, multicollinearity statistics show that LQ and FER accuracy were unique predictors of MSCS total (tolerance = 0.99–1.00, variance inflation factor = 1.00), suggesting that there is little statistical overlap between LQ and FER. Left-visual-field bias for facial emotion processing was independently predictive of increased social competency (*B* = −11.64, *p* =.004) and an increased FER accuracy was also independently predictive of increased social competency (*B* = 0.90, *p* <.001).

The theoretical approach to model construction was to examine the predictive effect of chimeric LQ onto MSCS subscales and second order factors. The addition of FER was treated as a fellow predictor or covariate, depending on the model tested. As such, MSCS variables were treated as endogenous, while chimeric LQ and FER were treated as exogenous.

Initially, several SEM models were tested to assure acceptable model fit. All models tested are elaborated upon here, with relevant model fit estimates for failed and successful models available in Table S4 found in Supplementary Information. The factor structure with two second order factors (Social Understanding and Social Responsiveness) described in Trevisan et al. (2018) was not able to converge. An exploratory model with gender as a multigroup factor was also conducted, displaying acceptable RMSEA but especially poor CFI and TLI (Table S4), likely indicating a mismatch with the theoretical model and the currently defined model (Shi et al., 2019). An analysis was also conducted at the level of factor subscale scores and FER acting as a covariate, which similarly displayed poor fit (Table S4). Two well-fitting models will be discussed below, one model at the item level and one model at the factor level, using totalled factor scores.

An item level SEM model tested the theoretical model with 77 MSCS items at the factor level predicted by chimeric LQ and FER (as covariate), with 348 participants and 385 free parameters. Initially, the model displayed acceptable fit. To improve model fit whilst also preserving the theoretical structure of the scale, a conservative approach was employed to remove items with spurious and high cross factor loadings. These included items from SM (19, 69), VCS (6, 7, 8, 63, 74). Additionally, the following items from the NSS (items 62 and 72) and SM (items 57 and 62) were constrained as covariates. After the modifications were made, the chi-square test of model fit was significant (χ^2^(2519) = 6687.09, *p* <.001), indicating a significant discrepancy between the model and the data. However, the indices of fit demonstrated that the model had good fit overall (RMSEA = 0.069, CFI = 0.948, TLI = 0.946). We also examined the residuals and found no major correlations in the residual matrix, as all residual correlations were below.5. Therefore, the model was considered to fit the data reasonably well.

The path estimates and beta values for the above SEM are available in the supplementary information (Table S5). The path diagram is also available in the supplementary information (Fig. S1). As expected, the relationship between FER and chimeric LQ was not significant, given the lack of a correlation between these variables noted above. In addition to the above analyses, the relationships between FER and the MSCS subscales are largely significant and present with stronger overall standardized β values than those where chimeric LQ act as a predictor. However, the directions of the parameter estimates were not in the expected direction given the above hierarchical regression results and may indicate that the model is not the good theoretical fit.

The SEM model at the factor level was conducted to include seven factor subscale total scores predicted by chimeric LQ and FER, with 348 participants and 54 free parameters (Table S4). This model displayed perfect fit, χ^2^(0) = 0.00, RMSEA = 0.000, TLI = 1.000, CFI = 1.000. Although these statistics are usually treated with caution (Kenny, 2024), it is noted that this model contrasts a rejected model due to poor fit (Model 4, Table S4) where FER was treated only as a covariate. Furthermore, the model here generally matches the theoretical explanation established by the above hierarchical regression models, where FER holds more predictive weight than chimeric LQ for the MSCS, but chimeric LQ does have a unique contribution.

**Fig. 3:**
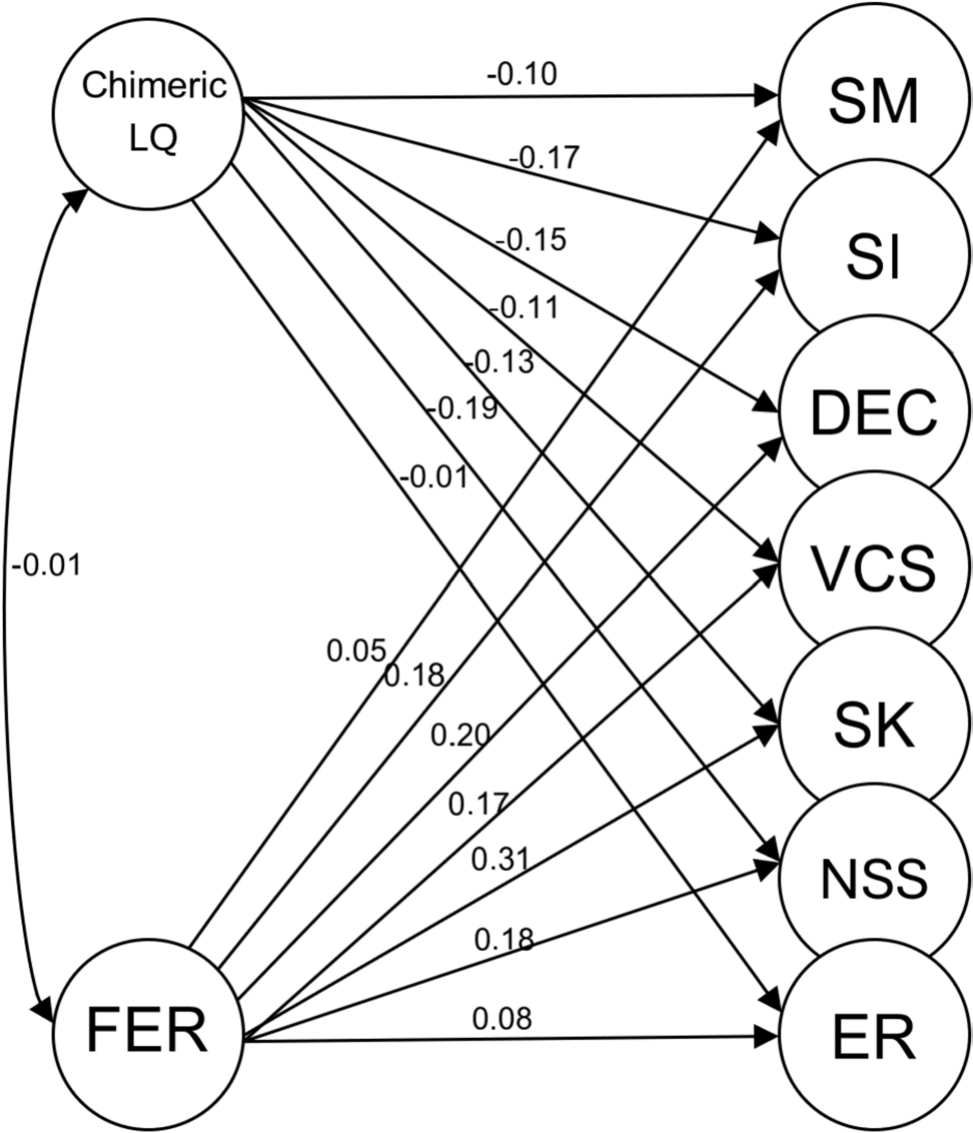
A path model derived from SEM demonstrating the path coefficients (β) for chimeric LQ (laterality quotient) and facial emotional recognition (FER) predicting multidimensional social competency scale (MSCS) subscales: Social Motivation (SM), Social Inferencing (SI), Demonstrating Empathic Concern (DEC), Social Knowledge (SK), Verbal Conversational Skills (VCS), Nonverbal Sending Skills (NSS), and Emotional Regulation (ER). Note that inter-factor correlations are removed for visual brevity. See Table S4 and Table S5 in Supplementary Information for goodness of fit statistics and parameter estimates

At the path level, the analyses suggest that chimeric LQ is a consistent predictor of a number of MSCS subscales, with the general direction suggesting a negative relationship and a trend towards preference for the left visual field. See Fig. 3 for path model and Table 3 for parameter estimates. The addition of FER as a co-predictor seems to also be consistent, with those higher in FER predicting stronger social skills, and especially strong in the SK subscale. For chimeric LQ, the strongest effects were revealed for NVSS and SI subscales.

**Table 3:** Parameter estimates for structural equation path model with chimeric LQ and FER as the predictor for MSCS subscales, conducted at the factor level

Predictor	Dependent	Estimate	SE	Β	β 95% CI	*p*
**Chimeric LQ**	SM	−0.105	0.055	−0.105	[−0.211, 0.002]	.057
	DEC	−0.155	0.056	−0.15	[−0.254, −0.047]	.005
	NSS	−0.194	0.056	−0.187	[−0.289, −0.085]	<.001
	SI	−0.179	0.056	−0.173	[−0.276, −0.07]	.001
	SK	−0.142	0.056	−0.133	[−0.234, −0.033]	.011
	VCS	−0.111	0.055	−0.109	[−0.213, −0.004]	.045
	ER	−0.01	0.055	−0.01	[−0.117, 0.098]	.859
**FER**	SM	0.05	0.055	0.05	[−0.057, 0.157]	.362
	DEC	0.202	0.056	0.196	[0.094, 0.298]	<.001
	NSS	0.191	0.056	0.184	[0.082, 0.287]	<.001
	SI	0.19	0.056	0.183	[0.081, 0.286]	<.001
	SK	0.334	0.058	0.314	[0.217, 0.41]	<.001
	VCS	0.178	0.056	0.174	[0.07, 0.278]	0.001
	ER	0.081	0.055	0.08	[−0.027, 0.187]	0.144

*SM* = social motivation; *DEC* = demonstrating empathic concern; *NSS* = nonverbal conversational skills; *SI* = social Inferencing; *SK* = social knowledge; *VCS* = verbal conversational skills; *ER = *emotional regulation

## Discussion

The purpose of the present work was to establish whether a relationship between social competencies and lateralisation of face processing could be observed. We expected that the strength of right-hemisphere dominance for face processing would be predictive of superior self-reported social competencies. This novel hypothesis was supported, with a behavioural index of face lateralisation, the laterality quotient (LQ), correlating significantly with overall social competencies (MSCS). Subsequently, structural equation modelling showed that LQ was best predicts social competency subscales relating to nonverbal sending skills, social inferencing, and empathy, although social knowledge and verbal conversational skills were related to a lesser extent. Importantly, given that the chimeric face task relies on processing emotional expressions from faces, we showed that these different social competencies were independent predictors of face lateralisation, over and above face emotion recognition performance.

Our findings are consistent with previous work that has highlighted the importance of the right hemisphere in face and social processing. A recent meta-analysis provides a causal link showing that while stroke patients demonstrated deficits in theory of mind and social perception regardless of the hemisphere affected, these were more pronounced for right than for left hemisphere damage (Adams et al., 2019). Hewetson et al. (2021) investigated the impact of these social cognitive deficits on relationships following a right-hemisphere stroke, finding the number of friends in a patients’ social network reduced, while the relationship quality was negatively impacted.

The correlation between right-hemisphere bias for face perception and social competencies that we report also finds support from previous studies utilising the chimeric face task. Stronger right-hemisphere bias on the chimeric task has previously been found to predict emotion recognition performance, which was already mentioned, is a component of social competencies (Watling & Damaskinou, 2020; Workman et al., 2006). Surprisingly, we did not replicate this, finding no correlation between LQ and face emotion recognition performance. One important factor that might account for this difference is that these previous studies examined children, where development of behavioural and neural mechanisms associated with face perception is not yet mature (Batty & Taylor, 2006; Cohen Kadosh et al., 2010; Pascalis et al., 2011). Furthermore, differences in face emotion recognition tasks could contribute to the different findings; for example, Workman et al. (2006) assessed recognition of emotions from cartoon images of faces and presentation of only the eye region of faces, whereas the current study used photos and videos of real actors. Nevertheless, the current findings extend this literature by suggesting that face lateralisation is associated with a broader range of social competencies, beyond the narrower subcomponent of social cognition involved in recognising facial expressions. Indeed, the demonstration of this association in a neurotypical sample is consistent with the autism literature, which similarly suggests that social-communication skills, as a core diagnostic feature of autism, is linked to a reduced bias in right-hemisphere face processing (Ashwin et al., 2005; Leung et al., 2015; Taylor et al., 2012).

Beyond the assessment of face emotion recognition, we are not aware of any previous research testing the link between face lateralisation and broader social competencies. One aspect of social cognition that has been considered relates to the complexity and differentiation of emotion information processing. Lane et al. (1995) used the Levels of Emotional Awareness Scale in which participants are provided with verbal descriptions of emotion-evoking situations involving two people. Participants responded to open-ended questions about how the self and the other person would feel. Scores on this measure were correlated with lateralisation for faces as measured with a chimeric face task. Emotional awareness appears to be a discrete construct that is not directly assessed with the MSCS, and is most closely linked to emotional intelligence, but also alexithymia and theory of mind (Lane & Smith, 2021; Scheerer et al., 2021). Nevertheless, given that emotion self-regulation, as well as how complex social situations are navigated, may be contingent on emotional awareness (Lane & Smith, 2021), our findings are strongly in support of and build on those reported earlier by Lane et al. (1995).

It is intriguing to consider how a functional organisational principle of the brain, namely the degree of reliance on the right hemisphere for face emotion perception, could be associated with such a broad construct as social competencies. These competencies encompass various social-cognitive abilities as well as overt behaviours, such as making eye-contact (Trevisan et al., 2018). In part, there is a clear link in that the ability to recognise facial displays of emotion is an important driver of how an individual engages in a social interaction (Horstmann, 2003). Because of the important developmental timeline for the maturation of cortical contributions to face processing, we suggest that the development of a right-hemisphere bias in face perception, itself a function of complex biological and environmental interactions (Johnson, 2011; Karmiloff-Smith, 1998), is likely to precede the development of the array of social competencies measured in the current study. Thus, although highly speculative from the current findings, we consider it more likely that the development of a stronger right-hemisphere bias in face perception contributes to the development of social competencies.

Exploratory SEM analyses suggested that the nonverbal sending skills, and social inferencing subscales, were best predicted by LQ. The nonverbal sending subscale includes items relating to the projection of non-verbal social cues (e.g., forced/awkward smiling and eye-contact) while the social inferencing subscale relates to the understanding of cues from a social partner (detecting sarcasm or judging another person’s mental state). The only subscales to not predict LQ scores were the emotion regulation and the social motivation subscales.

Thus, although it is quite remarkable that such a broad set of social competencies can predict face lateralisation, it is apparent that competencies relating to both understanding social cues, as well as the display of these social cues, are most associated with right hemispheric face lateralisation. This is consistent with the suggestion that the right hemisphere is more strongly linked to the ability to understand the intentions of other people, including the ability to discern dynamics and manage social interactions (Hecht, 2014) and also consistent with the notion that a successful social interaction is dependent in large part on observing and understanding that interaction. Indeed, the face—the focus of the current study—is a critical stimulus in allowing us to both convey, but also understand the intentions, emotions, as well as many other qualities of another person (Horstmann, 2003). Furthermore, the role of the right hemisphere has been linked with a broad set of skills relating to social cognition, including pro-social predispositions (Hecht, 2014), whereas specific regions, such as the right temporoparietal junction (TPJ), are recruited for higher level social perceptions, such as for attributing mental states (Krall et al., 2015; Saxe & Wexler, 2005) and social decision making (Bitsch et al., 2018).

It is necessary then to consider further how the organisation of face processing in the brain could be linked to social competencies. While it has been established that damage to the right hemisphere can impact on social cognition (Hewetson et al., 2021; Knox & Douglas, 2009), there are also various reports of a developmental disorder, likely a subset of those with nonverbal learning disability referred to as developmental right-hemisphere syndrome (Mammarella & Cornoldi, 2014; Semrud-Clikeman & Hynd, 1990; Tsur et al., 1995; Voeller, 1986). These children show a range of deficits but have been described as having poor social perception, emotional difficulties, an inability to display affect, and poor use of eye-contact (Weintraub & Mesulam, 1983). Together, the critical causal role of visual inputs to the right hemisphere during infancy on later face perception (Grand et al., 2003) and the evidence from developmental learning disabilities relating to the right hemisphere (Mammarella & Cornoldi, 2014; Voeller, 1986) allows for the possibility that natural but not developmentally impaired variation in the extent of preferential bias in face lateralisation contributes to population-wide variation in social competencies.

Thus far, our discussion has focussed on the importance of the right hemisphere in face processing (Adibpour et al., 2018; Caharel et al., 2009; de Heering & Rossion, 2015; Kanwisher et al., 1997; Workman et al., 2006). It is however important to note that this role is preferential, and, for example, a face selective region within the inferior temporal cortex known as the fusiform gyrus (Kanwisher et al., 1997) has been shown to have different functions bilaterally (Meng et al., 2012; Rossion et al., 2000). In the current study, we used a chimeric task requiring attention to emotional expressions; thus, further research could establish whether social competencies can be linked to specific aspects of face perception.

The current study has some limitations that should be acknowledged. Rather than a direct measure of the brain, a perceptual task was used to assess face lateralisation, and hence some caution is required when making inferences about brain organisation. However, previous research has demonstrated that both the asymmetry of fusiform gyrus activation (Yovel et al., 2008), and the contralateral visual-field bias of the right fusiform gyrus (Harrison & Strother, 2021) are strong and specific predictors of the left-visual-field bias measured by a chimeric face task. This suggests that the chimeric face task is a useful behavioural measure of brain lateralisation. We also note that a self-report, rather than an experimentally assessed, measure of social competencies was utilised. While not without limitations, self-report measures are widely accepted in psychological research (Paulhus & Vazire, 2007), and indeed as pointed out by Trevisan et al. (2018), the subjective viewpoint provided by a self-report scale offers novel insights not possible from other methods. Thus, it is important to note that the conclusions reported are limited to self-reported social competencies, and future work should test whether these findings are replicated with objective lab-based social skill assessment. It is also worth adding a note of caution regarding the small effect sizes that we report. Consequently, factors beyond social competencies account for most of the variance in face lateralisation (e.g., social anxiety is one candidate factor; Bourne & Vladeanu, 2011). Nevertheless, the correlation established here is highly significant, and our reported effect sizes are typical for social and developmental psychology studies (Weinerová et al., 2022). The current sample had a larger proportion of female participants which limits the ability to generalise the findings. Our analyses SEM analyses could not account for gender as a factor between LQ and social competency, and we do not have reason to think that the general findings only relate to women, although replication in a more balanced sample would help confirm this hypothesis.

A further consideration relates to our focus on the lateralisation of face processing, rather than emotion. As noted in the introduction, a right-hemisphere-bias has been established with chimeric face tasks that do not rely on facial emotion processing (Aljuhanay et al., 2010; Butler et al., 2005; Harrison & Strother, 2021; Yovel et al., 2008). Despite this, it remains possible that the association between chimeric LQ scores and social competencies is a function of the emotion, rather than the face, processing required for the chimeric task. Our observation that chimeric LQ is a predictor of LQ, independent of a face emotion recognition performance, gives some support for our interpretation that focusses on face processing. Finally, as pointed out by English et al. (2023) in the context of a review of hemispheric asymmetry of visuospatial attention in autism, a difference in face lateralisation could be due to a specific difference in brain lateralisation for face perception, or it could instead be due to a more general difference in lateralisation of visuospatial attention. As such, future research could investigate whether social competencies are best predicted by differences in lateralisation of face perception or in the lateralisation of other nonface stimuli, for example in word perception which is known to be preferentially processed in the left hemisphere (Gabay et al., 2017).

## Conclusions

The practical utility of functional brain asymmetries is understudied and remains controversial (Dräger et al., 2005; Rossion & Lochy, 2022; Vallortigara et al., 1999). Following the findings from a single study suggesting that a stronger right lateralisation of face processing predicted emotional awareness (Lane et al., 1995), we find evidence that this can be extended to a broader set of social competencies, in particular those relating to making social inferences and the communication of nonverbal social cues. Although face perception is a necessary component of successful social interactions, the link between a perceptual bias in face processing that reflects neural organisation (Yovel et al., 2008) and a broad construct encompassing how humans interact in social settings is intriguing and requires replication. These findings enhance understanding of fundamental aspect of affective neuroscience, illuminating the neural correlates of social competencies.

## Supplementary Information

Below is the link to the electronic supplementary material.

Supplementary file1 (DOCX 215 KB)

## Data Availability

The data that support the findings of this study are openly available in Figshare at https://figshare.com/s/5f3f7d93a016edd94b8c (authors deidentified).

## References

[CR1] Abbott, J. D., Cumming, G., Fidler, F., & Lindell, A. K. (2013). The perception of positive and negative facial expressions in unilateral brain-damaged patients: A meta-analysis. *Laterality,**18*(4), 437–459. 10.1080/1357650X.2012.703206 22849611 10.1080/1357650X.2012.703206

[CR2] Adams, A. G., Schweitzer, D., Molenberghs, P., & Henry, J. D. (2019). A meta-analytic review of social cognitive function following stroke. *Neuroscience and Biobehavioral Reviews,**102*, 400–416. 10.1016/j.neubiorev.2019.03.011 30922978 10.1016/j.neubiorev.2019.03.011

[CR3] Adibpour, P., Dubois, J., & Dehaene-Lambertz, G. (2018). Right but not left hemispheric discrimination of faces in infancy. *Nature Human Behaviour,**2*(1), 67–79. 10.1038/s41562-017-0249-4 30980049 10.1038/s41562-017-0249-4

[CR4] Adolphs, R., Damasio, H., Tranel, D., & Damasio, A. R. (1996). Cortical systems for the recognition of emotion in facial expressions. *The Journal of Neuroscience,**16*(23), 7678–7687. 10.1523/jneurosci.16-23-07678.1996 8922424 10.1523/JNEUROSCI.16-23-07678.1996 PMC6579085

[CR5] Aljuhanay, A., Milne, E., Burt, D. M., & Pascalis, O. (2010). Asymmetry in face processing during childhood measured with chimeric faces. *Laterality,**15*(4), 439–450. 10.1080/13576500902972823 19548168 10.1080/13576500902972823

[CR6] Alves, N. T., Fukusima, S. S., & Aznar-Casanova, J. A. (2008). Models of brain asymmetry in emotional processing. *Psychology & Neuroscience,**1*, 63–66.

[CR7] Anwyl-Irvine, A. L., Massonnié, J., Flitton, A., Kirkham, N., & Evershed, J. K. (2020). Gorilla in our midst: An online behavioral experiment builder. *Behavior Research Methods,**52*(1), 388–407. 31016684 10.3758/s13428-019-01237-x PMC7005094

[CR8] Ashwin, C., Wheelwright, S., & Baron-Cohen, S. (2005). Laterality biases to chimeric faces in Asperger syndrome: What is right about face-processing? *Journal of Autism and Developmental Disorders,**35*(2), 183–196. 10.1007/s10803-004-1997-3 15909405 10.1007/s10803-004-1997-3

[CR9] Batty, M., & Taylor, M. J. (2006). The development of emotional face processing during childhood. *Developmental Science,**9*(2), 207–220. 10.1111/j.1467-7687.2006.00480.x 16472321 10.1111/j.1467-7687.2006.00480.x

[CR10] Baumeister, R. F., & Leary, M. R. (1995). The need to belong: Desire for interpersonal attachments as a fundamental human motivation. *Psychological Bulletin,**117*(3), 497–529. 7777651

[CR11] Bitsch, F., Berger, P., Nagels, A., Falkenberg, I., & Straube, B. (2018). The role of the right temporo–parietal junction in social decision-making. *Human Brain Mapping,**39*(7), 3072–3085. 10.1002/hbm.24061 29582502 10.1002/hbm.24061 PMC6866486

[CR12] Borod, J. C. (1992). Interhemispheric and intrahemispheric control of emotion: A focus on unilateral brain damage. *Journal of Consulting and Clinical Psychology,**60*(3), 339–348. 10.1037/0022-006X.60.3.339 1619088 10.1037//0022-006x.60.3.339

[CR13] Bourne, V. J. (2008). Examining the relationship between degree of handedness and degree of cerebral lateralization for processing facial emotion. *Neuropsychology,**22*(3), 350–356. 10.1037/0894-4105.22.3.350 18444713 10.1037/0894-4105.22.3.350

[CR14] Bourne, V. J. (2010). How are emotions lateralised in the brain? Contrasting existing hypotheses using the chimeric faces test. *Cognition and Emotion,**24*(5), 903–911. 10.1080/02699930903007714

[CR15] Bourne, V. J., & Gray, D. L. (2011). One face or two? Contrasting different versions of the chimeric faces test. *Laterality: Asymmetries of Body, Brain and Cognition*. 10.1080/1357650X.2010.498119 10.1080/1357650X.2010.498119 21259158

[CR16] Bourne, V. J., & Vladeanu, M. (2011). Lateralisation for processing facial emotion and anxiety: Contrasting state, trait and social anxiety. *Neuropsychologia,**49*(5), 1343–1349. 10.1016/j.neuropsychologia.2011.02.008 21315746 10.1016/j.neuropsychologia.2011.02.008

[CR17] Butler, S., Gilchrist, I. D., Burt, D. M., Perrett, D. I., Jones, E., & Harvey, M. (2005). Are the perceptual biases found in chimeric face processing reflected in eye-movement patterns? *Neuropsychologia,**43*(1), 52–59. 10.1016/j.neuropsychologia.2004.06.005 15488905 10.1016/j.neuropsychologia.2004.06.005

[CR18] Caharel, S., d’Arripe, O., Ramon, M., Jacques, C., & Rossion, B. (2009). Early adaptation to repeated unfamiliar faces across viewpoint changes in the right hemisphere: Evidence from the N170 ERP component. *Neuropsychologia,**47*(3), 639–643. 10.1016/j.neuropsychologia.2008.11.016 19084547 10.1016/j.neuropsychologia.2008.11.016

[CR19] Cohen Kadosh, K., Cohen Kadosh, R., Dick, F., & Johnson, M. H. (2010). Developmental changes in effective connectivity in the emerging core face network. *Cerebral Cortex,**21*(6), 1389–1394. 10.1093/cercor/bhq215 21045001 10.1093/cercor/bhq215 PMC3094719

[CR20] Collier, J. (2020). *Applied Structural Equation Modeling using AMOS: Basic to Advanced Techniques* (1st ed). Routledge. 10.4324/9781003018414

[CR21] Dawel, A., Miller, E. J., Horsburgh, A., & Ford, P. (2022). A systematic survey of face stimuli used in psychological research 2000–2020. *Behavior Research Methods,**54*(4), 1889–1901. 10.3758/s13428-021-01705-3 34731426 10.3758/s13428-021-01705-3

[CR22] de Haan, M., & Nelson, C. A. (1999). Brain activity differentiates face and object processing in 6-month-old infants. *Developmental Psychology,**35*(4), 1113–1121. 10.1037/0012-1649.35.4.1113 10442879 10.1037//0012-1649.35.4.1113

[CR23] de Heering, A., & Rossion, B. (2015). Rapid categorization of natural face images in the infant right hemisphere. *eLife,**4*, Article e06564. 10.7554/eLife.06564 26032564 10.7554/eLife.06564 PMC4450157

[CR24] Dobs, K., Bülthoff, I., & Schultz, J. (2018). Use and usefulness of dynamic face stimuli for face perception studies—a review of behavioral findings and methodology [Mini Review]. *Frontiers in Psychology*, *Volume 9 - 2018*. 10.3389/fpsyg.2018.01355 10.3389/fpsyg.2018.01355 PMC6085596 30123162

[CR25] Downey, L. E., Mahoney, C. J., Buckley, A. H., Golden, H. L., Henley, S. M., Schmitz, N., Schott, J. M., Simpson, I. J., Ourselin, S., Fox, N. C., Crutch, S. J., & Warren, J. D. (2015). White matter tract signatures of impaired social cognition in frontotemporal lobar degeneration. *NeuroImage: Clinical,**8*, 640–651. 10.1016/j.nicl.2015.06.005 26236629 10.1016/j.nicl.2015.06.005 PMC4513187

[CR26] Dräger, B., Breitenstein, C., & Knecht, S. (2005). Rethinking brain asymmetries in humans. *Behavioral and Brain Sciences,**28*(4), 598–599. 10.1017/S0140525X05320103

[CR27] Dundas, E. M., Best, C. A., Minshew, N. J., & Strauss, M. S. (2012). A lack of left visual field bias when individuals with autism process faces. *Journal of Autism and Developmental Disorders,**42*(6), 1104–1111. 10.1007/s10803-011-1354-2 21986874 10.1007/s10803-011-1354-2 PMC3428133

[CR28] Dundas, E. M., Gastgeb, H., & Strauss, M. S. (2012). Left visual field biases when infants process faces: A comparison of infants at high- and low-risk for autism spectrum disorder. *Journal of Autism and Developmental Disorders,**42*(12), 2659–2668. 10.1007/s10803-012-1523-y 22527700 10.1007/s10803-012-1523-y PMC3408549

[CR29] English, M. C. W., Maybery, M. T., & Visser, T. A. W. (2023). A review of behavioral evidence for hemispheric asymmetry of visuospatial attention in autism. *Autism Research,**16*(6), 1086–1100. 10.1002/aur.2956 37243312 10.1002/aur.2956

[CR30] Faul, F., Erdfelder, E., Buchner, A., & Lang, A.-G. (2009). Statistical power analyses using G* power 3.1: Tests for correlation and regression analyses. *Behavior Research Methods,**41*(4), 1149–1160. 19897823 10.3758/BRM.41.4.1149

[CR31] Gabay, Y., Dundas, E., Plaut, D., & Behrmann, M. (2017). Atypical perceptual processing of faces in developmental dyslexia. *Brain and Language,**173*, 41–51. 10.1016/j.bandl.2017.06.004 28624595 10.1016/j.bandl.2017.06.004 PMC13074411

[CR32] Grand, R. L., Mondloch, C. J., Maurer, D., & Brent, H. P. (2003). Expert face processing requires visual input to the right hemisphere during infancy. *Nature Neuroscience,**6*(10), 1108–1112. 10.1038/nn1121 12958600 10.1038/nn1121

[CR33] Harrison, M. T., & Strother, L. (2021). Does face-selective cortex show a left visual field bias for centrally-viewed faces? *Neuropsychologia,**159*, Article 107956. 10.1016/j.neuropsychologia.2021.107956 34265343 10.1016/j.neuropsychologia.2021.107956 PMC8373204

[CR34] Hecht, D. (2014). Cerebral lateralization of pro- and anti-social tendencies. *Experimental Neurobiology,**23*(1), 1–27. 10.5607/en.2014.23.1.1 24737936 10.5607/en.2014.23.1.1 PMC3984952

[CR35] Hewetson, R., Cornwell, P., & Shum, D. H. K. (2021). Relationship and social network change in people with impaired social cognition post right hemisphere stroke. *American Journal of Speech-Language Pathology,**30*(2S), 962–973. 10.1044/2020_AJSLP-20-00047 33621120 10.1044/2020_AJSLP-20-00047

[CR36] Horstmann, G. (2003). What do facial expressions convey: Feeling states, behavioral intentions, or actions requests? *Emotion,**3*(2), 150–166. 10.1037/1528-3542.3.2.150 12899416 10.1037/1528-3542.3.2.150

[CR37] Indersmitten, T., & Gur, R. C. (2003). Emotion processing in chimeric faces: Hemispheric asymmetries in expression and recognition of emotions. *The Journal of Neuroscience,**23*(9), 3820. 10.1523/JNEUROSCI.23-09-03820.2003 12736352 10.1523/JNEUROSCI.23-09-03820.2003 PMC6742199

[CR38] Innes, B. R., Burt, D. M., Birch, Y. K., & Hausmann, M. (2016). A leftward bias however you look at it: Revisiting the emotional chimeric face task as a tool for measuring emotion lateralization. *Laterality,**21*(4–6), 643–661. 10.1080/1357650X.2015.1117095 26710248 10.1080/1357650X.2015.1117095

[CR39] Jacques, C., d’Arripe, O., & Rossion, B. (2007). The time course of the inversion effect during individual face discrimination. *Journal of Vision,**7*(8), 3–3. 10.1167/7.8.3 17685810 10.1167/7.8.3

[CR40] Johnson, M. H. (2005). Subcortical face processing. *Nature Reviews Neuroscience,**6*(10), 766–774. 10.1038/nrn1766 16276354 10.1038/nrn1766

[CR41] Johnson, M. H. (2011). Interactive specialization: A domain-general framework for human functional brain development? *Developmental Cognitive Neuroscience,**1*(1), 7–21. 10.1016/j.dcn.2010.07.003 22436416 10.1016/j.dcn.2010.07.003 PMC6987575

[CR42] Johnson, M. H., Dziurawiec, S., Ellis, H., & Morton, J. (1991). Newborns’ preferential tracking of face-like stimuli and its subsequent decline. *Cognition,**40*(1–2), 1–19. 1786670 10.1016/0010-0277(91)90045-6

[CR43] Jones, D. E., Greenberg, M., & Crowley, M. (2015). Early social-emotional functioning and public health: The relationship between kindergarten social competence and future wellness. *American Journal of Public Health,**105*(11), 2283–2290. 10.2105/ajph.2015.302630 26180975 10.2105/AJPH.2015.302630 PMC4605168

[CR44] Kanwisher, N., McDermott, J., & Chun, M. M. (1997). The fusiform face area: A module in human extrastriate cortex specialized for face perception. *The Journal of Neuroscience,**17*(11), 4302–4311. 10.1523/jneurosci.17-11-04302.1997 9151747 10.1523/JNEUROSCI.17-11-04302.1997 PMC6573547

[CR45] Karmiloff-Smith, A. (1998). Development itself is the key to understanding developmental disorders [review]. *Trends in Cognitive Sciences,**2*(10), 389–398. 10.1016/s1364-6613(98)01230-3 21227254 10.1016/s1364-6613(98)01230-3

[CR46] Kenny, D. (2024). *SEM: Measuring model fit*. https://davidakenny.net/cm/fit.htm

[CR47] Knox, L., & Douglas, J. (2009). Long-term ability to interpret facial expression after traumatic brain injury and its relation to social integration. *Brain and Cognition,**69*(2), 442–449. 10.1016/j.bandc.2008.09.009 18951674 10.1016/j.bandc.2008.09.009

[CR48] Krall, S. C., Rottschy, C., Oberwelland, E., Bzdok, D., Fox, P. T., Eickhoff, S. B., Fink, G. R., & Konrad, K. (2015). The role of the right temporoparietal junction in attention and social interaction as revealed by ALE meta-analysis. *Brain Structure and Function,**220*(2), 587–604. 10.1007/s00429-014-0803-z 24915964 10.1007/s00429-014-0803-z PMC4791048

[CR49] Lane, R. D., Kivley, L. S., Du Bois, M. A., Shamasundara, P., & Schwartz, G. E. (1995). Levels of emotional awareness and the degree of right hemispheric dominance in the perception of facial emotion. *Neuropsychologia,**33*(5), 525–538. 10.1016/0028-3932(94)00131-8 7637851 10.1016/0028-3932(94)00131-8

[CR50] Lane, R. D., & Smith, R. (2021). Levels of emotional awareness: Theory and measurement of a socio-emotional skill. *Journal of Intelligence,**9*(3), 42. 10.3390/jintelligence9030042 34449662 10.3390/jintelligence9030042 PMC8395748

[CR51] Leppänen, J. M., & Nelson, C. A. (2009). Tuning the developing brain to social signals of emotions. *Nature Reviews Neuroscience,**10*(1), 37–47. 10.1038/nrn2554 19050711 10.1038/nrn2554 PMC2976651

[CR52] Leung, R. C., Pang, E. W., Cassel, D., Brian, J. A., Smith, M. L., & Taylor, M. J. (2015). Early neural activation during facial affect processing in adolescents with autism spectrum disorder. *NeuroImage: Clinical,**7*, 203–212. 10.1016/j.nicl.2014.11.009 25610782 10.1016/j.nicl.2014.11.009 PMC4300004

[CR53] Levy, J., Trevarthen, C., & Sperry, R. W. (1972). Perception of bilateral chimeric figures following hemispheric deconnexion. *Brain,**95*(1), 61–78. 10.1093/brain/95.1.61 5023091 10.1093/brain/95.1.61

[CR54] Li, C.-H. (2016). Confirmatory factor analysis with ordinal data: Comparing robust maximum likelihood and diagonally weighted least squares. *Behavior Research Methods,**48*(3), 936–949. 10.3758/s13428-015-0619-7 26174714 10.3758/s13428-015-0619-7

[CR55] Lundqvist, D., Flykt, A., & Öhman, A. (1998). The Karolinska directed emotional faces - KDEF. In. CD ROM from Department of Clinical Neuroscience, Psychology section, Karolinska Institute, ISBN 91-630-7164-9.

[CR56] Mammarella, I. C., & Cornoldi, C. (2014). An analysis of the criteria used to diagnose children with nonverbal learning disability (NLD). *Child Neuropsychology,**20*(3), 255–280. 10.1080/09297049.2013.796920 23705673 10.1080/09297049.2013.796920

[CR57] Meng, M., Cherian, T., Singal, G., & Sinha, P. (2012). Lateralization of face processing in the human brain. *Proceedings of the Royal Society B: Biological Sciences,**279*(1735), 2052–2061. 10.1098/rspb.2011.1784 10.1098/rspb.2011.1784 PMC3311882 22217726

[CR58] Najt, P., Bayer, U., & Hausmann, M. (2013). Models of hemispheric specialization in facial emotion perception—A reevaluation. *Emotion,**13*(1), 159–167. 10.1037/a0029723 22906088 10.1037/a0029723

[CR59] Pascalis, O., de Martin Viviés, X., Anzures, G., Quinn, P. C., Slater, A. M., Tanaka, J. W., & Lee, K. (2011). Development of face processing. *WIREs Cognitive Science,**2*(6), 666–675. 10.1002/wcs.146 22039564 10.1002/wcs.146 PMC3203018

[CR60] Paulhus, D. L., & Vazire, S. (2007). The self-report method. In *Handbook of research methods in personality psychology.* (pp. 224-239). The Guilford Press.

[CR61] Peltola, M. J., Yrttiaho, S., & Leppänen, J. M. (2018). Infants’ attention bias to faces as an early marker of social development. *Developmental Science,**21*(6), Article e12687. 10.1111/desc.12687 29971869 10.1111/desc.12687

[CR62] Rogers, L. J., Zucca, P., & Vallortigara, G. (2004). Advantages of having a lateralized brain. *Proceedings of the Royal Society of London. Series B, Biological Sciences,**271*(suppl_6), S420–S422. 10.1098/rsbl.2004.0200 10.1098/rsbl.2004.0200 PMC1810119 15801592

[CR63] Rosseel, Y. (2012). Lavaan: An R package for structural equation modeling. *Journal of Statistical Software,**48*(1), 1–36. 10.18637/jss.v048.i02

[CR64] Rossion, B., Caldara, R., Seghier, M., Schuller, A. M., Lazeyras, F., & Mayer, E. (2003). A network of occipito-temporal face-sensitive areas besides the right middle fusiform gyrus is necessary for normal face processing. *Brain,**126*(11), 2381–2395. 10.1093/brain/awg241 12876150 10.1093/brain/awg241

[CR65] Rossion, B., Dricot, L., Devolder, A., Bodart, J.-M., Crommelinck, M., de Gelr, B., & Zoontjes, R. (2000). Hemispheric asymmetries for whole-based and part-based face processing in the human fusiform gyrus. *Journal of Cognitive Neuroscience,**12*(5), 793–802. 10.1162/089892900562606 11054921 10.1162/089892900562606

[CR66] Rossion, B., Joyce, C. A., Cottrell, G. W., & Tarr, M. J. (2003). Early lateralization and orientation tuning for face, word, and object processing in the visual cortex. *NeuroImage,**20*(3), 1609–1624. 10.1016/j.neuroimage.2003.07.010 14642472 10.1016/j.neuroimage.2003.07.010

[CR67] Rossion, B., & Lochy, A. (2022). Is human face recognition lateralized to the right hemisphere due to neural competition with left-lateralized visual word recognition? A critical review. *Brain Structure and Function,**227*(2), 599–629. 10.1007/s00429-021-02370-0 34731327 10.1007/s00429-021-02370-0

[CR68] Saxe, R., & Wexler, A. (2005). Making sense of another mind: The role of the right temporo-parietal junction. *Neuropsychologia,**43*(10), 1391–1399. 10.1016/j.neuropsychologia.2005.02.013 15936784 10.1016/j.neuropsychologia.2005.02.013

[CR69] Scheerer, N. E., Boucher, T. Q., & Iarocci, G. (2021). Alexithymia is related to poor social competence in autistic and nonautistic children. *Autism Research,**14*(6), 1252–1259. 10.1002/aur.2485 33616273 10.1002/aur.2485

[CR70] Scherf, K. S., Behrmann, M., Humphreys, K., & Luna, B. (2007). Visual category-selectivity for faces, places and objects emerges along different developmental trajectories. *Developmental Science,**10*(4), F15–F30. 10.1111/j.1467-7687.2007.00595.x 17552930 10.1111/j.1467-7687.2007.00595.x

[CR71] Semrud-Clikeman, M., & Hynd, G. W. (1990). Right hemisphere dysfunction in nonverbal learning disabilities: Social, academic, and adaptive functioning in adults and children. *Psychological Bulletin,**107*(2), 196–209. 10.1037/0033-2909.107.2.196 2181523 10.1037/0033-2909.107.2.196

[CR72] Shi, D., Lee, T., & Maydeu-Olivares, A. (2019). Understanding the model size effect on SEM fit indices. *Educational and Psychological Measurement,**79*(2), 310–334. 10.1177/0013164418783530 30911195 10.1177/0013164418783530 PMC6425088

[CR73] Taylor, S., Workman, L., & Yeomans, H. (2012). Abnormal patterns of cerebral lateralisation as revealed by the universal chimeric faces task in individuals with autistic disorder. *Laterality,**17*(4), 428–437. 10.1080/1357650X.2010.521751 22690895 10.1080/1357650X.2010.521751

[CR74] Trevisan, D. A., Tafreshi, D., Slaney, K. L., Yager, J., & Iarocci, G. (2018). A psychometric evaluation of the Multidimensional Social Competence Scale (MSCS) for young adults. *PLoS One,**13*(11), Article e0206800. 10.1371/journal.pone.0206800 30388171 10.1371/journal.pone.0206800 PMC6214554

[CR75] Tsur, V. G., Shalev, R. S., Manor, O., & Amir, N. (1995). Developmental right-hemisphere syndrome: Clinical spectrum of the nonverbal learning disability. *Journal of Learning Disabilities,**28*(2), 80–86. 10.1177/002221949502800202 7884301

[CR76] Vallortigara, G., Rogers, L. J., & Bisazza, A. (1999). Possible evolutionary origins of cognitive brain lateralization. *Brain Research Reviews,**30*(2), 164–175. 10.1016/S0165-0173(99)00012-0 10525173 10.1016/s0165-0173(99)00012-0

[CR77] van der Schalk, J., Hawk, S. T., Fischer, A. H., & Doosje, B. (2011). Moving faces, looking places: Validation of the Amsterdam Dynamic Facial Expression Set (ADFES). *Emotion,**11*(4), 907–920. 10.1037/a0023853 21859206 10.1037/a0023853

[CR78] Vladeanu, M., Monteith-Hodge, E. M., & Bourne, V. J. (2012). Strength of lateralisation for processing facial emotion in relation to autistic traits in individuals without autism. *Laterality,**17*(4), 438–452. 10.1080/1357650X.2010.513385 21452096 10.1080/1357650X.2010.513385

[CR79] Voeller, K. K. (1986). Right-hemisphere deficit syndrome in children. *American Journal of Psychiatry,**143*(8), 1004–1009. 10.1176/ajp.143.8.1004 3728713 10.1176/ajp.143.8.1004

[CR80] Wang, Y. A., & Rhemtulla, M. (2021). Power analysis for parameter estimation in structural equation modeling: A discussion and tutorial. *Advances in Methods and Practices in Psychological Science,**4*(1), 2515245920918253. 10.1177/2515245920918253

[CR81] Watling, D., & Damaskinou, N. (2020). Children’s facial emotion recognition skills: Longitudinal associations with lateralization for emotion processing. *Child Development,**91*(2), 366–381. 10.1111/cdev.13188 30521072 10.1111/cdev.13188

[CR82] Weinerová, J., Szűcs, D., & Ioannidis, J. P. A. (2022). Published correlational effect sizes in social and developmental psychology. *Royal Society Open Science,**9*(12), Article 220311. 10.1098/rsos.220311 36569230 10.1098/rsos.220311 PMC9768465

[CR83] Weintraub, S., & Mesulam, M.-M. (1983). Developmental learning disabilities of the right hemisphere: Emotional, interpersonal, and cognitive components. *Archives of Neurology,**40*(8), 463–468. 10.1001/archneur.1983.04210070003003 6870605 10.1001/archneur.1983.04210070003003

[CR84] Workman, L., Chilvers, L., Yeomans, H., & Taylor, S. (2006). Development of cerebral lateralisation for recognition of emotions in chimeric faces in children aged 5 to 11. *Laterality,**11*(6), 493–507. 10.1080/13576500600724963 16966239 10.1080/13576500600724963

[CR85] Yager, J., & Iarocci, G. (2013). The development of the Multidimensional Social Competence Scale: A standardized measure of social competence in autism spectrum disorders. *Autism Research,**6*(6), 631–641. 10.1002/aur.1331 24108618 10.1002/aur.1331

[CR86] Yovel, G., Tambini, A., & Brandman, T. (2008). The asymmetry of the fusiform face area is a stable individual characteristic that underlies the left-visual-field superiority for faces. *Neuropsychologia,**46*(13), 3061–3068. 10.1016/j.neuropsychologia.2008.06.017 18639566 10.1016/j.neuropsychologia.2008.06.017

