# Supplementary material for: Right-hemisphere lateralisation evidenced from the chimeric face task predicts self-reported social competencies
Source: Cogn Affect Behav Neurosci. 2025 Dec 10;26(3):1008–21. doi: 10.3758/s13415-025-01378-x (PMC13260032; doi:10.3758/s13415-025-01378-x)
Supplement: Supplementary file 1 — Supplementary file1 (DOCX 215 KB) [file 13415_2025_1378_MOESM1_ESM.docx]

**Supplementary Information**

*Static vs Dynamic Face Emotion Recognition*

We used both static and dynamic face stimuli. The correlations between FER accuracy (static, dynamic, and combined) and the LQ, MSCS, and their subscales were all similar, suggesting that dynamic FER did not explain a greater amount of variance in LQ or social competency measures than static FER (see Table S1).

Table S1

Correlations Between Face Emotion Recognition and Laterality Quotient, and MSCS Scores.

|  | Combined FER | Dynamic FER | Static FER |
| --- | --- | --- | --- |
| Chimeric LQ | -.028 | -.012 | -.039 |
| MSCS - Total Score | .256^***^ | .214^***^ | .239^***^ |
| MSCS Factor 1: Social Responsiveness | .191^***^ | .163^**^ | .176^****^ |
| MSCS Factor 2 Social Understanding / Emotion Regulation | .255^**^ | .211^***^ | .241^***^ |
| Social Motivation | .081 | .084 | .059 |
| Social Inferencing | .214^***^ | .181^***^ | .198^***^ |
| Empathic Concern | .200^***^ | .167^**^ | .187^***^ |
| Social Knowledge | .349^***^ | .312^***^ | .306^***^ |
| Verbal Conversational Skills | .194^***^ | .165^**^ | .178^***^ |
| Nonverbal Sending Skills | .206^***^ | .161^**^ | .204^***^ |
| Emotion Regulation | .094 | .054 | .113^*^ |

Note: * < 0.05; ** < 0.01; *** < 0.001

*Analysis of emotions selected for face tasks*

We ran further analyses to examine whether the difference in emotions used between the Chimeric face task (angry, fear, happy, sad) and the FER task (angry, fear, happy, sad, disgust, surprise, pride, contempt) could be influential. Thus, we calculated a second FER score this time only analysing the angry, fear, happy, and sad expressions of emotion. The correlation between the FER-8 (i.e., 8 emotions) and the FER-4 (i.e., 4 emotions) was very strong, *r*(346) = .769, *p* < .001, indicating both measures assess largely the same construct.

In Table S2, we show the correlations for the FER-8 and FER-4 against the Chimeric LQ and the various MSCS scores. Given that the LQ did not correlate with either FER measure, and also that the MSCS scores provided very consistent correlations, though slightly higher for FER-8 than for FER-4, we decided to use the FER-8 measure for the main analyses.

Table S2

Correlations Between FER Tasks Using Either 4 or 8 Emotions with Laterality Quotient, and MSCS Scores.

|  | FER-8 emotions | FER-4 emotions |
| --- | --- | --- |
| Chimeric LQ | -.028 | -.046 |
| MSCS - Total Score | .256^***^ | .221^***^ |
| MSCS Factor 1: Social Responsiveness | .191^***^ | .179^***^ |
| MSCS Factor 2 Social Understanding / Emotion Regulation | .255^***^ | .208^***^ |
| Social Motivation | .081 | .071 |
| Social Inferencing | .214^***^ | .171^**^ |
| Empathic Concern | .200^***^ | .186^***^ |
| Social Knowledge | .349^***^ | .270^***^ |
| Verbal Conversational Skills | .194^***^ | .169^**^ |
| Nonverbal Sending Skills | .206^***^ | .199^***^ |
| Emotion Regulation | .094 | .080 |

Note: * < 0.05; ** < 0.01; *** < 0.001

*Check for gender effects*

Due to concerns that gender differences in face emotion processing or social competency could influence results, we ran analyses to check if gender appeared as a potential confounding variable in our main analyses. Independent samples T tests were used to explore gender differences on all characteristics (see Table S3). Due to the small number of participants identifying as non-binary we did include this group in the following where gender differences were examined.

There was no significant difference in visual field bias (LQ) between genders, with both males and females demonstrating a significant left-hemifield visual field bias. This aligns with a recent online study using a Chimeric face task (Smekal et al., 2022; but see also Bourne, 2005, Innes et al., 2016).

Males scored slightly worse than females on FER accuracy (< 3% difference). Interestingly, there was no gender difference for the total social competency score (MSCS). Females also scored moderately higher than males on demonstrating empathic concern, social knowledge, and non-verbal sending skills sub-scales, while males scored higher on emotional regulation, and there were no gender differences on the social inferencing, social motivation, and verbal conversation skills subscales. Trevisan et al. (2018) had identified 2 factors from the subscales, and we found that females scored moderately higher than males on the Social Responsiveness factor while no gender difference emerged for the Social Understanding factor. We also calculated the effect sizes for gender comparisons based on the data from Trevisan et al. and found in the main very similar findings (see Table S3).

Given there were no gender differences in LQ or MSCS, while noting a small difference for FER, in the main analyses we did not consider gender further.

Table S3

Descriptive Statistics and Gender Differences for Participant Characteristics, Laterality Quotient, FER Accuracy, and MSCS Scores

|  | Male  *N =* 82 | | Female  *N =* 256 | |  |  |
| --- | --- | --- | --- | --- | --- | --- |
|  | *M* | *SD* | *M* | *SD* | Cohen’s *d*  Current study | Cohen’s *d*  Trevisan study |
| Age | 21.12 | 2.88 | 20.46 | 2.76 | 0.24 |  |
| Chimeric LQ^†^ | -0.20 | 0.39 | -0.19 | 0.40 | 0.03 |  |
| Facial Emotion Recognition Accuracy | 56.96 | 8.30 | 59.46 | 8.55 | 0.30^*^ |  |
| **MSCS Total** | 286.40 | 30.68 | 291.94 | 29.78 | 0.18 |  |
| **Social understanding factor^††^** | 167.20 | 15.84 | 165.29 | 17.94 | 0.11 |  |
| Social motivation | 37.28 | 6.96 | 38.69 | 6.92 | 0.20 | 0.208^***^ |
| Demonstrating empathic | 42.37 | 6.63 | 45.29 | 5.49 | 0.51^***^ | 0.631^***^ |
| concern |  |  |  |  |  |  |
| Non-verbal sending skills | 41.29 | 6.51 | 43.05 | 6.11 | 0.28^*^ | 0.636^**^* |
| **Social responsiveness factor^††^** | 120.89 | 16.55 | 127.10 | 15.68 | 0.39^**^ |  |
| Social inferencing | 42.39 | 5.23 | 42.22 | 5.75 | 0.03 | 0.008 |
| Social knowledge | 46.59 | 4.07 | 48.48 | 4.30 | 0.44^***^ | 0.440^***^ |
| Verbal conversation skills | 38.46 | 5.58 | 38.73 | 6.20 | 0.04 | 0.28^***^ |
| Emotional regulation | 39.52 | 6.08 | 35.66 | 6.85 | 0.58^***#^ | 0.194^**#^ |

Note. ^*^ *p* < .05, ^**^ *p* < .01, ^***^ *p* < .001, based on between genders independent samples T-test, with females scoring higher than males, except where indicated by #.
† Chimeric LQ scores closer to -1 indicate increased left visual field bias and scores closer to +1 indicate increased right visual field bias.
†† The 2-factor MSCS model is derived from Trevisan, et al. (2018). The social understanding factor consists of social motivation, demonstrating empathic concern and non-verbal sending skills subscales. The social responsiveness factor consists of social inferencing, social knowledge, verbal conversation skills and emotion regulation subscales

Bolding denotes higher level factors.

Table S4

Comparison of SEM Goodness of Fit Statistics for 5 Multidimensional Social Competency Scale (MSCS) Models with Chimeric LQ as the Predictor

| Description | df | χ² | p | RMSEA | CFI | TFI |
| --- | --- | --- | --- | --- | --- | --- |
| Model 1: Item level, Second order factors^†^ | - | - | - | - | - | - |
| Model 2: Item level, Gender as a multigroup factor, with first order subscales only | 5936 | 12402.415 | <.001 | 0.080 | .545 | .528 |
| Model 3: Item level, first order subscales only | 2519 | 6687.09 | <.001 | 0.069 | .948 | .946 |
| Model 4: Factor level, FER as covariate | 7 | 39.093 | <.001 | 0.118 | .965 | .822 |
| Model 5: Factor level, FER as predictor | 0 | 0 | - | 0.000 | 1.000 | 1.000 |

^†^ Model failed to converge

Table S5

Parameter Estimates for Structural Equation Path Model with Chimeric LQ as the Predictor for MSCS subscales and FER as a Co-Predictor, Conducted at the Item level

| **Predictor** | **Dependent** | **Estimate** | **SE** | **β** | **β 95% CI** | **p** |
| --- | --- | --- | --- | --- | --- | --- |
| **Chimeric LQ** | SM | 0.139 | 0.086 | 0.091 | [-0.018, 0.2] | .105 |
|  | DEC | 0.140 | 0.067 | 0.115 | [0.01, 0.22] | .037 |
|  | NVCS | 0.243 | 0.082 | 0.159 | [0.055, 0.262] | .003 |
|  | SI | -0.213 | 0.090 | -0.133 | [-0.242, -0.024] | .018 |
|  | SK | 0.156 | 0.062 | 0.133 | [0.032, 0.235] | .012 |
|  | VCS | 0.193 | 0.120 | 0.096 | [-0.021, 0.212] | .107 |
|  | ER | -0.019 | 0.064 | -0.018 | [-0.135, 0.099] | .760 |
|  | SM | -0.005 | 0.004 | -0.065 | [-0.182, 0.053] | .280 |
| **FER** | DEC | -0.012 | 0.003 | -0.210 | [-0.32, -0.1] | <.001 |
|  | NVCS | -0.018 | 0.004 | -0.249 | [-0.352, -0.147] | <.001 |
|  | SI | 0.018 | 0.004 | 0.237 | [0.144, 0.33] | <.001 |
|  | SK | -0.020 | 0.004 | -0.358 | [-0.463, -0.253] | <.001 |
|  | VCS | -0.023 | 0.006 | -0.239 | [-0.349, -0.129] | <.001 |
|  | ER | -0.006 | 0.003 | -0.119 | [-0.228, -0.01] | 0.049 |

Note: This describes Model 4 from Table S4 above.

Figure S1

Path Model for Item Level Structural Equation Model with Chimeric LQ Predicting MSCS Subscales, with FER as a Predictor

**
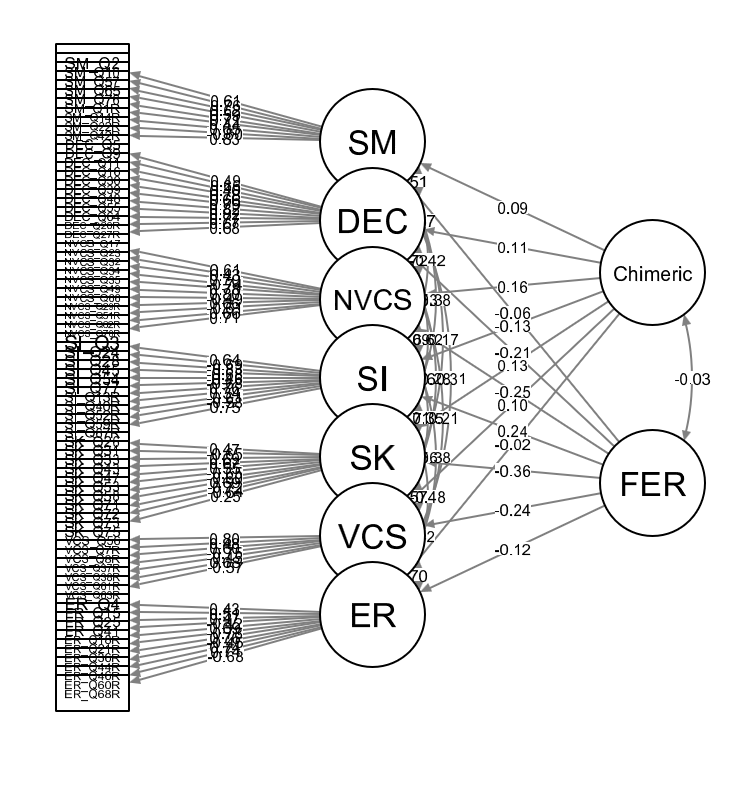
**

**References**

Bourne, V. J. (2005). Lateralised processing of positive facial emotion: Sex differences in strength of hemispheric dominance. *Neuropsychologia*, *43*(6), 953-956.

Innes, B. R., Burt, D. M., Birch, Y. K., & Hausmann, M. (2016). A leftward bias however you look at it: Revisiting the emotional chimeric face task as a tool for measuring emotion lateralization. Laterality: Asymmetries of Body, *Brain and Cognition*, *21*(4-6), 643- 661.

Smekal, V., Burt, D. M., Kentridge, R. W., & Hausmann, M. (2022). Emotion lateralization in a graduated emotional chimeric face task: An online study. Neuropsychology, 36(5), 443.Bourne, V. J. (2005). Lateralised processing of positive facial emotion: Sex differences in strength of hemispheric dominance. *Neuropsychologia*, *43*(6), 953-956.

Trevisan, D. A., Tafreshi, D., Slaney, K. L., Yager, J., & Iarocci, G. (2018). A psychometric evaluation of the Multidimensional Social Competence Scale (MSCS) for young adults. *PLoS One*, *13*(11), e0206800. https://doi.org/10.1371/journal.pone.0206800
